# Incidence of chikungunya virus infections among Kenyan children with neurological disease, 2014–2018: A cohort study

**DOI:** 10.1371/journal.pmed.1003994

**Published:** 2022-05-12

**Authors:** Doris K. Nyamwaya, Mark Otiende, Lilian Mwango, Symon M. Kariuki, Berrick Otieno, Donwilliams O. Omuoyo, George Githinji, Barnes S. Kitsao, Henry K. Karanja, John N. Gitonga, Zaydah R. de Laurent, Alun Davies, Salim Mwarumba, Charles N. Agoti, Samuel M. Thumbi, Mainga M. Hamaluba, Charles R. Newton, Philip Bejon, George M. Warimwe

**Affiliations:** 1 KEMRI-Wellcome Trust Research Programme, Nairobi, Kenya; 2 Paul G Allen School for Global Animal Health, Washington State University, Washington, United States of America; 3 Institute of Immunology and Infection Research, University of Edinburgh, Edinburgh, United Kingdom; 4 Center for Epidemiological Modelling and Analysis, Institute of Tropical and Infectious Diseases, University of Nairobi, Nairobi, Kenya; 5 Centre for Tropical Medicine and Global Health, University of Oxford, Oxford, United Kingdom; Mahidol-Oxford Tropical Medicine Research Unit, THAILAND

## Abstract

**Background:**

Neurological complications due to chikungunya virus (CHIKV) infection have been described in different parts of the world, with children being disproportionately affected. However, the burden of CHIKV-associated neurological disease in Africa is currently unknown and given the lack of diagnostic facilities in routine care it is possible that CHIKV is an unrecognized etiology among children with encephalitis or other neurological illness.

**Methods and findings:**

We estimated the incidence of CHIKV infection among children hospitalized with neurological disease in Kilifi County, coastal Kenya. We used reverse transcriptase polymerase chain reaction (RT-PCR) to systematically test for CHIKV in cerebrospinal fluid (CSF) samples from children aged <16 years hospitalized with symptoms of neurological disease at Kilifi County Hospital between January 2014 and December 2018. Clinical records were linked to the Kilifi Health and Demographic Surveillance System and population incidence rates of CHIKV infection estimated. There were 18,341 pediatric admissions for any reason during the 5-year study period, of which 4,332 (24%) had CSF collected. The most common clinical reasons for CSF collection were impaired consciousness, seizures, and coma (47%, 22%, and 21% of all collections, respectively). After acute investigations done for immediate clinical care, CSF samples were available for 3,980 admissions, of which 367 (9.2%) were CHIKV RT-PCR positive. Case fatality among CHIKV-positive children was 1.4% (95% CI 0.4, 3.2). The annual incidence of CHIKV-associated neurological disease varied between 13 to 58 episodes per 100,000 person-years among all children <16 years old. Among children aged <5 years, the incidence of CHIKV-associated neurological disease was 77 per 100,000 person-years, compared with 20 per 100,000 for cerebral malaria and 7 per 100,000 for bacterial meningitis during the study period. Because of incomplete case ascertainment due to children not presenting to hospital, or not having CSF collected, these are likely minimum estimates. Study limitations include reliance on hospital-based surveillance and limited CSF sampling in children in coma or other contraindications to lumbar puncture, both of which lead to under-ascertainment of incidence and of case fatality.

**Conclusions:**

In this study, we observed that CHIKV infections are relatively more common than cerebral malaria and bacterial meningitis among children hospitalized with neurological disease in coastal Kenya. Given the wide distribution of CHIKV mosquito vectors, studies to determine the geographic extent of CHIKV-associated neurological disease in Africa are essential.

## Abstract 2

In a cohort study, Doris K. Nyamwaya, Mark Otiende, and colleagues investigate the incidence of chikungunya virus infection-associated neurological illness among children younger than 16 years of age in Kilifi County, Kenya, between 2014-2018.

## Author summary

**Why was this study done?:**

- Chikungunya virus (CHIKV) was first discovered in East Africa and is a known cause of neurological illness, especially in young children.
- CHIKV-associated neurological illness has been reported in many parts of the world, but there have been no reports to our knowledge of CHIKV-associated neurological illness in children from countries in Africa.
- This study aimed to estimate the incidence of CHIKV-associated neurological illness in coastal Kenya where CHIKV infections in children are relatively common.

**What did the researchers do and find?:**

- We conducted clinical surveillance at the Kilifi County Hospital in coastal Kenya over a 5-year period (2014 to 2018), during which there were 18,341 admissions aged <16 years. Of these, 4,332 had a lumbar puncture for cerebrospinal fluid (CSF) analysis due to symptoms of neurological illness.
- CHIKV was detected in CSF samples from 367 (9.2%) of 3,980 hospitalized children with samples available after acute investigations done for immediate clinical care. With linkage to demographic surveillance, we estimated an incidence of 77 CHIKV-associated neurological disease cases/100,000 person-years for children aged <5 years, which is higher than the incidence for bacterial meningitis (7 per 100,000 person-years) and cerebral malaria (20 per 100,000 person-years) during the same period.

**What do these findings mean?:**

- Our findings suggest a high burden of CHIKV infections among children hospitalized with neurological disease in coastal Kenya.
- Surveillance for CHIKV in CSF samples has not been undertaken systematically in Africa and should now be an urgent priority.

## Introduction

Chikungunya virus (CHIKV) is a positive sense RNA virus of the *Alphavirus* genus that was first discovered in Tanzania in 1953 [1]. CHIKV is transmitted between humans by *Aedes aegypti* and *Ae*. *albopictus* mosquitoes [2], which have facilitated its rapid global spread and the numerous chikungunya fever (CHIKF) epidemics reported to date [3]. In adults, CHIKF is characterized by abrupt onset of fever and debilitating muscle and joint pain, following an incubation period of about 2 to 10 days [4]. Most infections are self-limiting, but joint and musculoskeletal pain may persist for months to years in some individuals [4]. However, unlike adults, young children rarely present with musculoskeletal symptoms but are more likely to be hospitalized with CHIKV-associated neurological disease [5–10]. No specific therapeutics or licensed vaccines are available for CHIKF, and, in the absence of pathognomonic clinical features, confirmatory diagnosis relies on laboratory detection of CHIKV in clinical samples [4].

CHIKV transmission is widely reported in Africa [3], with endemic febrile disease being particularly common in children [11–14]. In 2004, one of the largest CHIKF epidemics on record began in coastal Kenya and spread rapidly along the East African coast and to islands in the Indian Ocean [15–17]. During that epidemic, severe neurological manifestations of CHIKF were described in children in La Reunion [6,18], including disease in neonates suggesting mother-to-child virus transmission [19,20]. CHIKV-associated neurological disease has since been reported in other geographical settings [5,8], besides Africa.

Recent analysis identified a high, previously unrecognized, burden of endemic CHIKF in children presenting at outpatient primary care facilities in coastal Kenya [14]. Here, we used an established ward surveillance at Kilifi County Hospital (KCH) on the north coast of Kenya, to estimate the incidence of CHIKV-associated neurological disease among children in this setting. Cerebrospinal fluid (CSF) samples taken for clinical reasons at the hospital are routinely stored and clinical records linked to the Kilifi Health and Demographic Surveillance System (KHDSS), allowing estimation of population incidence rates [21]. We therefore systematically tested these stored CSF samples to establish whether evidence of infection by CHIKV is common among children admitted to hospital with neurological illness in coastal Kenya.

## Methods

### Clinical surveillance

KCH is a referral public hospital in rural coastal Kenya with approximately 4,000 pediatric admissions annually. The hospital’s catchment area includes the approximately 290,000 KHDSS residents under continuous demographic surveillance (for births, deaths, and migration) who account for approximately 40% to 50% of all admissions in Kilifi County and are enumerated during household census rounds conducted every 4 months [21]. The KHDSS covers an area of 891 km^2^, has a male:female ratio of 90:100, and a population growth rate of 2.8%. Mortality rate in children aged <5 and 5 to 14 years is 5.4 deaths and 2.4 deaths per 1,000 person-years of observation (PYO) [22]. Morbidity events recorded at the KCH are linked in real time with the demographic events of the KHDSS residents by means of unique person identifiers. A few other private hospitals and lower-level facilities offer inpatient services for KHDSS residents, but they are not part of this study. Malaria transmission is endemic in the demographic surveillance area with seasonal rains occurring in April to June and October to December [23]. The pediatric service at KCH includes 2 wards; a 70-bed general ward and a 15-bed high dependency unit (HDU) staffed by research clinicians and nurses. The HDU admits children with serious illness requiring more intensive monitoring and management but has no ventilation facilities or renal replacement therapy. Electronic case records are kept for all admissions including demographics, vital signs, clinical history, and examination, as well as routine laboratory investigations such as malaria blood slides and blood and CSF culture. Each patient is assigned a discharge diagnosis by the attending clinician following review of the notes and results. All data are linked to the KHDSS database in real time by means of unique person identifiers.

For this study, all children aged <16 years whose illness required sampling and analysis of CSF were eligible for inclusion. The decision to collect CSF was made by clinicians. The same senior clinicians oversaw care throughout the period of surveillance. Where acute coma was present, the medical team considered the risks of lumbar puncture versus clinical benefits of diagnostic information from a clinical management perspective without reference to research considerations. Where clinically appropriate, delayed lumbar punctures were conducted after a period of treatment. Neuroimaging was not available on site during the period of surveillance. After investigations done for immediate clinical care, an aliquot of CSF was stored at −80°C and used for CHIKV testing by reverse transcriptase polymerase chain reaction (RT-PCR) as described below. Written informed consent to use stored clinical samples for research was provided by parents or guardians of all study participants. Ethical approval for use of the clinical and demographic surveillance framework was provided by the Kenya Medical Research Institute Scientific and Ethics Review Unit (SSC No. 3296). Our approach was to screen all stored CSF for CHIKV and link these data to demographic surveillance (KHDSS) for incidence estimation as part of a larger program of work aimed at estimating the case burden of arboviral illnesses in coastal Kenya [14,24]. This study is reported as per the Strengthening the Reporting of Observational Studies in Epidemiology (STROBE) guideline (S1 STROBE Checklist). This manuscript was submitted for publication with permission from the Director of the Kenya Medical Research Institute.

### Detection of CHIKV infection

Total RNA was isolated from 100 μl of each CSF sample using TRIzol Reagent (Thermo Fisher, USA). A published primer–probe set targeting the CHIKV nonstructural protein 1 (nsP1) region [25] was then used to detect CHIKV viral RNA using the Taqman Fast Virus RT-PCR kit (Thermo Fisher) on a 7500 Real-Time PCR System (Thermo Fisher Scientific, USA) in a 10-μl reaction volume comprising 3 μl of 4x Taqman Fast Virus 1-step master mix, 5 μl RNA, and primers (CHIKV 874, CHIKV 961) and probe (CHIKV 899) [25] at final concentrations of 800 nM and 200 nM, respectively. RT-PCR conditions were reverse transcription at 50°C for 5 minutes, RT inactivation/initial activation at 95°C for 20 seconds, and 45 cycles of denaturation at 95°C for 3 seconds and annealing/extension at 60°C for 30 seconds [14]. This assay is highly specific and can detect CHIKV in viral RNA isolated from blood samples obtained from febrile children in this setting, including confirmation by whole viral genome sequencing [14]. A positive result was defined as a cycle threshold (Ct) value of <40. Viral RNA from a cultured CHIKV isolate (GenBank accession: MT526796) was used as positive control, and nuclease-free water was used as negative control [14]. Assay specificity was also confirmed by genome sequencing of a subset of 13 CSF samples (7 RT-PCR positive and 6 RT-PCR negative) with CHIKV genomes only obtained from the 7 RT-PCR-positive samples (S4 Fig).

### Statistical analysis

We did not have a prospective analysis plan, and the exercise of screening stored CSF was prompted by the identification of CHIKV in the CSF of a child with prolonged coma. The analysis that followed the identification of further positive results was primarily descriptive (Tables 1 and 2), and the significance testing using Poisson regression was then altered to negative binomial regression (which accommodates overdispersed data) in response to peer review. All children hospitalized between 2014 and 2018 and whose illness required sampling and analysis of CSF were included in the analysis. Demographic and clinical features were compared between CHIKV-positive and CHIKV-negative admissions using chi-squared test for categorical variables and Mann–Whitney U test for analysis of the number of days hospitalized as a continuous variable. Cerebral malaria was defined as admission with a *Plasmodium falciparum* parasite density >2,500/μl of blood and a Blantyre Coma Score (BCS) <3, while impaired consciousness was defined as BCS 3 or 4 [23,26]. Acute bacterial meningitis was defined as either (i) a positive CSF bacterial culture or latex agglutination test; or (ii) bacteremia accompanied by a CSF-to-blood glucose ratio <0.1; or (iii) bacteremia accompanied by CSF white blood cell count ≥50 × 10^6^ cells/L [27,28]. We required evidence of bacteria in CSF or in blood in the case definition of meningitis to maximize specificity, since there are no data on glucose or CSF cell counts for CHIKV infection in our setting.

**Table 1:** Demographic and clinical features of patients screened for CHIKV infection.

	CHIKV positive (*N =* 367)	CHIKV negative (*N* = 3,613)	*P* value
**Sex–no. (%)**			0.39
Female	161 (43.9)	1,501 (41.5)	
**Age group–no. (%)**			0.39
<3 months	148 (40.3)	1,641 (45.4)	
3 to <12 months	36 (9.8)	306 (8.5)	
1 to <5 years	124 (33.8)	1,164 (32.2)	
5 to <10 years	48 (13.1)	399 (11.0)	
10 to 15 years	11 (3.0)	103 (2.8)	
**Year of admission–no. (%)**			<0.001
2014	68 (18.5)	918 (25.4)	
2015	91 (24.8)	895 (24.8)	
2016	144 (39.2)	639 (17.7)	
2017	33 (9.0)	457 (12.6)	
2018	31 (8.4)	704 (19.5)	
**Season–no. (%)**			0.32
January–March	92 (25.1)	986 (27.3)	
April–June	110 (30.0)	995 (27.5)	
July–September	90 (24.5)	792 (21.9)	
October–December	75 (20.4)	840 (23.2)	
**Admission characteristics**			
Duration (days) of hospitalization (median, IQR)	3 (2, 6)	4 (2, 7)	0.26
Needed blood transfusion (no., %)	22 (6.0)	185 (5.1)	0.46
**General symptoms–no. (%)**			
Fever	279 (76.0)	2,683 (74.3)	0.46
Vomiting	57 (15.5)	548 (15.2)	0.85
Cough	60 (16.3)	629 (17.4)	0.61
Diarrhea	20 (5.4)	251 (6.9)	0.28
Jaundice	25 (6.8)	312 (8.6)	0.23
Wasting	15 (4.1)	150 (4.1)	0.95
Joint pain	2 (0.5)	10 (0.3)	0.37
Irritability	16 (4.4)	236 (6.5)	0.10
Rash	1 (0.3)	34 (0.9)	0.19
Deep breathing	29 (7.9)	356 (9.8)	0.23
Shock	2 (0.5)	33 (0.9)	0.47
Lymphadenopathy	3 (0.8)	27 (0.7)	0.88
**Neurological symptoms–no. (%)** ^**#**^			
History of seizures	56 (15.3)	469 (13.0)	0.22
Seizures during current illness*	179 (48.8)	1,632 (45.2)	0.19
Headache	19 (5.2)	144 (4.0)	0.27
Bulging fontanelle	10 (2.7)	70 (1.9)	0.31
Neck stiffness	3 (0.8)	81 (2.2)	0.07
Agitation	27 (7.4)	239 (6.6)	0.59
Prostration	56 (15.3)	574 (15.9)	0.75
Impaired consciousness	165 (45.0)	1,718 (47.5)	0.34
Coma	82 (22.3)	791 (21.9)	0.84
**Laboratory investigations–no. (%)** ^**†**^			
CSF-to-blood glucose ratio <0.67	63 (29.6)	681 (28.7)	0.80
CSF protein > 0.45 g/L	154 (43.7)	1,607 (46.5)	0.31
CSF leukocyte count >5/μL	52 (14.7)	567 (16.2)	0.47
CSF turbidity	9 (2.7)	158 (4.8)	0.09
HIV positive	8 (2.8)	73 (2.6)	0.80
Bacteremia	16 (4.4)	179 (5.0)	0.62
Malaria slide positive	90 (24.7)	815 (22.7)	0.39
Malaria parasite density (>2,500/μL)	57 (15.6)	531 (14.8)	0.67
Impaired renal function (creatinine >80 μmol/L)	89 (26.5)	894 (28.4)	0.45
Severe anemia (Hb <5 g/dL)	13 (3.6)	108 (3.0)	0.56
Hypoglycemia (blood glucose <2.2 mmol/l)	15 (6.8)	243 (9.9)	0.12
Thrombocytopenia (platelets <159 × 10^3^/μL)	81 (22.4)	767 (21.5)	0.71
Leukopenia (WBC count<3.9 x 10^3^/μL)	5 (1.4)	53 (1.5)	0.87
Lymphopenia (Lymphocyte count<1.7 × 10^3^/μL)	37 (10.2)	293 (8.2)	0.19

^**#**^Symptoms are not mutually exclusive; some patients had seizures and prostration, neck stiffness and agitation, and other overlaps in symptoms.

*Refers to at least 1 seizure in the last 24 hours. The frequency of multiple seizures (>2 in the last 24 hours) was comparable between CHIKV-positive and CHIKV-negative patients (35.2% versus 30.2%, *p* = 0.18); the bulk of seizures were generalized (84.2% and 79.3% for CHIKV-negative and CHIKV-positive children, respectively).

^**†**^Sample sizes for each variable do not always add up to the total number (N) for each group due to missing data. Analysis was only performed in those with data available. Missing data are summarized in S1 Table. *P* values are from chi-squared test comparing variables, except duration of hospitalization for which a Mann–Whitney U test was used.

CHIKV, chikungunya virus; CSF, cerebrospinal fluid; Hb, hemoglobin; HIV, human immunodeficiency virus; IQR, interquartile range; WBC, white blood cell count.

**Table 2:** Incidence of CHIKV-associated neurological disease among children aged <16 years within the KHDSS during the study period (2014–2018).

		Chikungunya (*N* = 207)			Cerebral Malaria (*N* = 68)			Acute Bacterial Meningitis (*N* = 22)		
Variable	Categories	n/PYO	Incidence/100,000 (95% CI)	IRR (95% CI)	n/PYO	Incidence/100,000 (95% CI)	IRR (95% CI)	n/PYO	Incidence/100,000 (95% CI)	IRR (95% CI)
**Sex**	**Female**	95/341,753	27.8 (22.7–34)	1	33/341,899	9.7 (6.9–13.6)	1	7/341,753	2 (1–4.3)	1
	**Male**	112/349,835	32 (26.6–38.5)	1.2 (0.9–1.5)	35/350,019	10 (7.2–13.9)	1.0 (0.6–1.7)	15/349,835	4.3 (2.6–7.1)	2.1 (0.8–5.1)
**Age**	**<3 months**	77/10,837	710.5 (568.3–888.4)	1	0/10,853	-	-	12/10,851	110.6 (62.8–194.7)	1
	**3 to <12 months**	19/33,700	56.4 (36–88.4)	0.1 (0–0.1)***	2/33,755	5.9 (1.5–23.7)	1	2/33,750	5.9 (1.5–23.7)	0.1 (0.0–0.2)***
	**1 to <5 years**	77/179,432	42.9 (34.3–53.7)	0.1 (0–0.1)***	42/179,650	23.4 (17.3–31.6)	3.9 (1–16.3)	1/179,697	0.6 (0.1–4)	0 (0–0)***
	**5 to <10 years**	28/227,125	12.3 (8.5–17.9)	0 (0–0)***	20/227,148	8.8 (5.7–13.6)	1.5 (0.3–6.4)	3/227,227	1.3 (0.4–4.1)	0 (0–0)***
	**10 to 15 years**	6/240,495	2.5 (1.1–5.6)	0 (0–0)***	4/240,512	1.7 (0.6–4.4)	0.3 (0.1–1.5)	4/240,519	1.7 (0.6–4.4)	0 (0–0)***
**Year**	**2014**	41/138,881	29.52 (21.7–40.1)	1	15/138,891	10.8 (6.5–17.9)	1	4/138,897	2.9 (1.1–7.7)	1
	**2015**	50/138,008	36.2 (27.5–47.8)	1.2 (0.8–1.9)	24/138,042	17.4 (11.7–25.9)	1.6 (0.8–3.1)	6/138,062	4.3 (2–9.7)	1.5 (0.4–5.3)
	**2016**	79/136,770	57.8 (46.3–72)	2.0 (1.3–2.9)**	12/136,847	8.8 (5–15.4)	0.8 (0.4–1.7)	5/136,877	4.9 (1.5–8.8)	1.3 (0.3–4.7)
	**2017**	18/138,861	13 (8.2–20.6)	0.4 (0.3–0.8)*^a^	7/138,965	5 (2.4–10.6)	0.5 (0.2–1.2)	0/138,998	-	-
	**2018**	19/139,067	13.7 (8.7–21.4)	0.5 (0.3–0.8)*^b^	10/139,172	7.2 (3.9–13.4)	0.7 (0.3–1.5)	7/139,209	5 (2.4–10.5)	1.7 (0.5–6.0)
**Season**	**January–March**	56/172,895	32.4 (24.9–42.1)	1	18/172,967	10.4 (6.6–16.5)	1	5/172,996	2.9 (1.2–6.9)	1
	**April–June**	54/173,137	31.2 (23.9–40.7)	0.9 (0.3–2.3)	15/173,216	8.7 (5.2–14.4)	0.8 (0.4–1.8)	9/173,245	5.2 (2.7–10)	1.8 (0.6–5.4)
	**July–September**	53/172,957	30.6 (23.4–40.1)	0.6 (0.2–1.6)	18/173,043	10.4 (6.6–16.5)	1.0 (0.4–2.2)	3/173,076	1.7 (0.6–5.4)	0.6 (0.1–2.5)
	**October–December**	44/172,599	25.5 (19–34.3)	0.6 (0.2–1.6)	17/172,692	9.8 (6.1–15.8)	1.0 (0.4–2.1)	5/172,726	2.9 (1.2–7)	1 (0.3–3.5)

CHIKV, chikungunya virus; IRR, unadjusted incidence rate ratio; KHDSS, Kilifi Health and Demographic Surveillance System; PYO: person-years observed.

*P* values

*^a^*p* = 0.004

*^b^*p* = 0.006

**p < 0.001

***p < 0.0001.

Incidence of CHIKV infection in the KHDSS was calculated as the number of CHIKV-positive cases divided by total PYO using time-to-event analysis methods in a longitudinal dataset. The denominator (PYO) or risk time was calculated as the duration of time from the latest of birth or in-migration or study start date to the earliest of (i) CHIKV-positive hospitalization; (ii) 16th birthday; (iii) date of last out-migration from the KHDSS; (iv) date of death; and (v) end of the study (31 December 2018). Periods of absence from the KHDSS area, where an individual migrated out of the KHDSS and in-migrated back were excluded from the PYO. We conducted a sensitivity analysis handling in/out migration in 2 different ways, which had a trivial impact on the estimates of incidence (S3 Table). For comparison, incidence estimates for cerebral malaria and acute bacterial meningitis were also calculated similarly to the incidence of CHIKV infection. Total PYO in the 3 incidence calculations would be slightly different because time-to-hospitalization with CHIKV, cerebral malaria, or bacterial meningitis was not the same. Incidence rate ratios comparing rates between sociodemographic variables were estimated using negative binomial regression models. All analyses were carried out in STATA/IC version 15.1 (StataCorp College Station, Texas, USA). We also examined the geographical distribution of cases of CHIKV infection, cerebral malaria, meningitis, and other admissions across the KHDSS area by mapping (using QGIS version 3.10) the geolocations of homes where the children lived at the time of admission.

## Results

**Fig 1:**
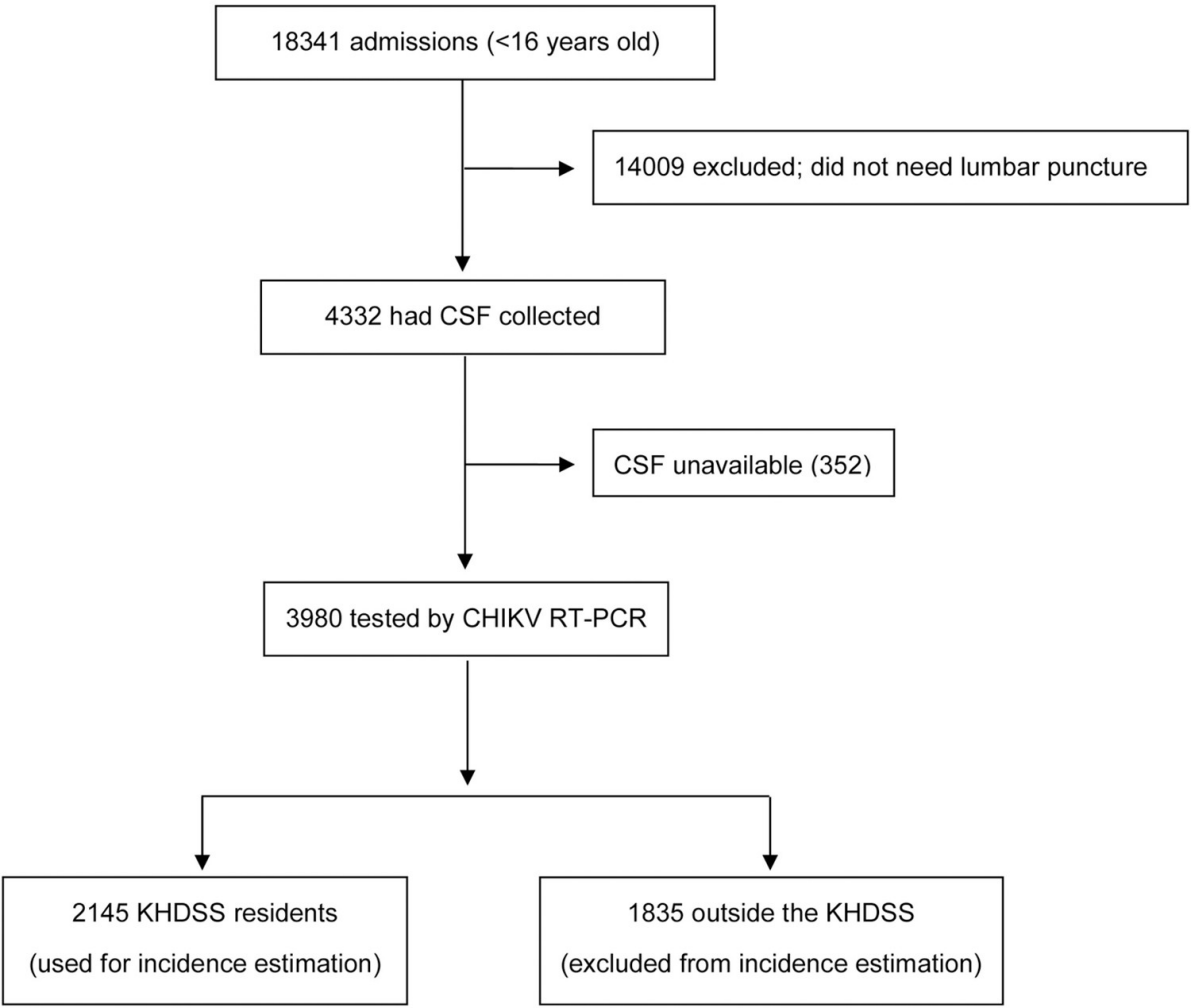
Flow of study participants. CHIKV, chikungunya virus; CSF, cerebrospinal fluid; KHDSS, Kilifi Health and Demographic Surveillance System; RT-PCR, reverse transcriptase polymerase chain reaction.

Between January 2014 and December 2018, 18,341 children aged <16 years were admitted at KCH, of whom 4,332 (24%) had CSF collected for routine investigations (Fig 1). The most common clinical indications for CSF collection were coma, impaired consciousness, and seizures, which together accounted for 90% of all CSF collections (S1 Fig). Similar proportions of children with these lumbar puncture indications had CSF collected across the 5-year study period, suggesting a consistent pattern of clinical practice throughout (S2 Fig). After acute investigations were done for immediate clinical care, stored CSF was available for 3,980 (92%) of the 4,332 admissions and these were screened for CHIKV infection (Fig 1).

Of the 3,980 admissions among children aged <16 years, 367 (9.2%, 95% CI 8.3, 10.2) were CHIKV RT-PCR positive. Most of these CHIKV infections (308 of the 367; 84%) were in children aged under 5 years (Table 1). RT-PCR assay Ct values for CHIKV-positive samples showed no correlation with age (Spearman’s rho = −0.09, *p* = 0.07). CHIKV RT-PCR positivity was highest in 2016 (18%), when an epidemic was reported in Kenya [29], and ranged between 4% to 9% in the other years (Table 1). CHIKV infection showed no association with HIV, bacteremia, or malaria parasitemia at the time of admission (Table 1). Further, the distribution of clinical history and symptoms recorded at admission and laboratory investigations undertaken to inform clinical care was similar for CHIKV-positive and CHIKV-negative children (Table 1). Among children below 3 months of age, the majority (1,352/1,789; 75%) of CSF samples were taken from newborns in the first week of life in whom we observed high CHIKV positivity rates (8.7%, 95% CI 7.3, 10.3: S2 Table) suggesting mother-to-child CHIKV transmission [30].

Overall mortality among all 18,341 admissions aged <16 years was 9.0% (95% CI 8.6, 9.4; 1,653 deaths). Mortality among the 4,332 admissions that had CSF collected during the study period (Fig 1) was 3.2% (95% CI 2.7, 3.8; 139 deaths), compared with 10.8% (95% CI 10.3, 11.3; 1,514 deaths) in those where CSF was not collected (S3 Fig). A similar pattern was observed among newborns in the first week of life, where overall mortality was 17.7% (95% CI 16.5, 18.8), whereas mortality in those whose CSF was collected was 2.5% (39 deaths) compared with 25.3% (773 deaths) in those where CSF was not collected.

Overall case fatality among CHIKV-positive children was 1.4% (95% CI 0.4, 3.2; 5 deaths of 367 CHIKV-positive children; 2 newborns, 2 aged 2 years and 1 aged 8 years) and 3.2% (95% CI 2.6, 3.8; 115 deaths) among CHIKV-negative children (Table 1).

**Fig 2:**
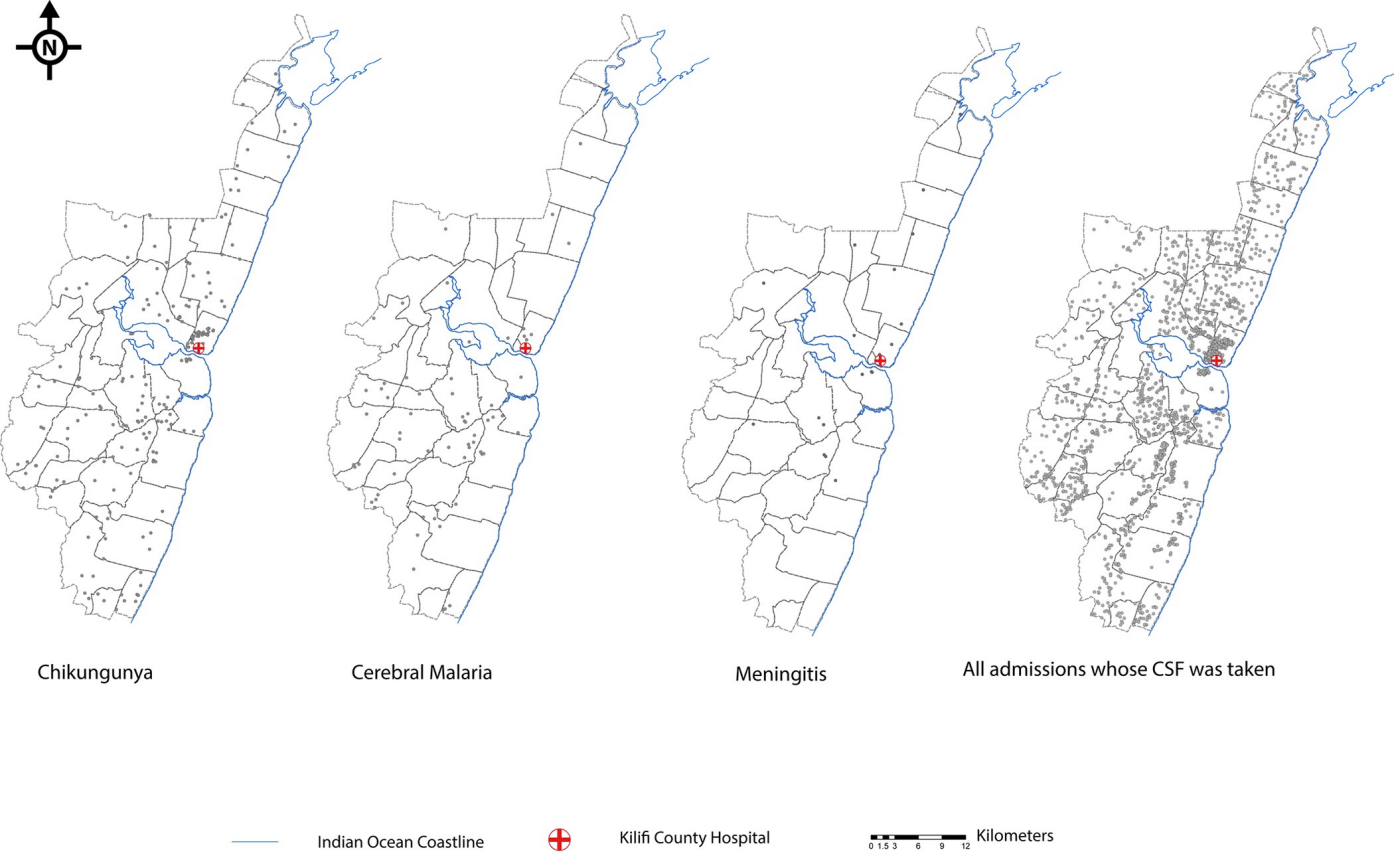
Distribution of children resident within the KHDSS that were admitted with neurological disease during the study period (2014–2018). Each point on the KHDSS map represents a child’s residential coordinates. The distribution of CHIKV-associated neurological cases is shown in comparison with that for children with cerebral malaria and meningitis or for all pediatric admissions that had CSF available for CHIKV RT-PCR screening. (Source: own elaboration using shapefiles and data from KEMRI-Wellcome Trust Research Programme). CHIKV, chikungunya virus; KHDSS, Kilifi Health and Demographic Surveillance System; CSF, cerebrospinal fluid; RT-PCR, reverse transcriptase polymerase chain reaction.

To estimate the incidence of CHIKV-associated neurological disease, we observed a total of 207 CHIKV RT-PCR positive admissions among KHDSS residents (out of the 367 resident and nonresident cases presenting to the hospital; Fig 2). The total risk time contributed during the 5-year study period by children aged <16 years was 691,588 person-years; Table 2). The overall incidence of CHIKV-associated neurological disease within the KHDSS was 30 per 100,000 person-years (95% CI 26.1, 34.3). Disease incidence was highest during the 2016 epidemic, but a high number of presentations with CHIKV infection were also detected in other years (Table 2). CHIKV-associated neurological disease cases were distributed throughout the KHDSS area (Fig 2). A strong inverse relationship was observed between the incidence of CHIKV infection and age, estimated at 77 per 100,000 person-years in all children aged <5 years and 7 per 100,000 person-years among children aged ≥5 years (Table 2). During the same period, we calculated the corresponding incidences of cerebral malaria and bacterial meningitis in children aged <5 years to be 20 per 100,000 and 7 per 100,000 person-years, respectively (Table 2). The corresponding incidence of cerebral malaria and bacterial meningitis in children aged ≥5 years was 5 per 100,000 and 1 per 100,000 person-years, respectively (Table 2).

## Discussion

There are currently no data on the burden of CHIKV-associated neurological disease in Africa, to the best of our knowledge. In this study, we aimed to estimate the incidence of CHIKV-associated neurological illness in coastal Kenya, through screening of stored CSF samples from children admitted with neurological illness between 2014 and 2018 and linkage to demographic surveillance. CHIKV viral RNA was detected in approximately 9% of all CSF samples in a county referral hospital. We observed CHIKV infections during and outside an epidemic year suggesting endemic CHIKV transmission in keeping with recent findings from community-level surveillance in the same setting [14]. The risk of CHIKV infection was highest in infants with disease being rare in older children, consistent with acquisition of immunity [14]. CHIKV infections were common among newborns, within the first week of life, suggesting mother-to-child virus transmission as has been observed by others [19,30].

The age-related risk of CHIKV-associated neurological disease has previously been observed in a landmark study in La Reunion Island where disease incidence was highest in young infants and in adults aged >65 years [6]. With the linkage to demographic surveillance, we estimate an incidence of 77 CHIKV-positive admissions per 100,000 person-years among children aged <5 years. This incidence is higher than recent incidences for bacterial meningitis, which we estimate at 7 per 100,000 person-years, or for invasive bacterial diseases such as 37 per 100,000 for invasive salmonellosis or 3 per 100,000 for invasive pneumococcal disease [27,31], and almost 4 times the incidence of cerebral malaria (20 per 100,000) in the same age group [23]. Because of incomplete case ascertainment due to children not presenting to hospital, or not having CSF collected, these are likely minimum estimates.

Approximately 92% of the 4,332 children that needed CSF collected for routine clinical investigations had meningism, coma, seizures, or mild reductions in BCS that did not meet the threshold for coma. The mortality among CHIKV-positive children was low, despite coma in 22% and depressed levels of consciousness in 47%. The decision to collect CSF via lumbar puncture was based on clinical priorities rather than research criteria. Even in those without obvious indications for CSF collection, the illness must have appeared sufficiently significant to clinicians to justify hospital admission and lumbar puncture. No specific antiviral treatment is available for CHIKV infection. However, most children with neurological illness will receive prolonged antibiotics in the absence of a specific diagnosis. Diagnosis would therefore improve antibiotic stewardship and avoid treatment costs. Furthermore, given the good prognosis that we observed associated with CHIKV, a diagnosis may allow some reassurance to be given to parents regarding outcome. However, the sickest patients are likely to die before CSF can be collected as clinicians are less likely to collect CSF in the first 24 hours when a patient presents in acute coma. During the 5-year period of monitoring 632 children with coma did not have CSF collected, of whom 248 (i.e., 39.2%) died, compared to 530 children with coma who did have CSF collected, 20 of whom died (i.e., 3.7%) (see S3 Fig). Children with coma may not have CSF collected acutely if clinicians are concerned about raised intracranial pressure, and on clinical recovery clinicians or families may decide to forego lumbar puncture. Our data suggest that children in whom CSF is not collected are at high risk of death, and it is therefore possible that CHIKV is a cause of mortality among these children. Future studies using PCR on serum or immunoglobulin M (IgM) serology against CHIKV will help address this. In addition, postdischarge follow-up of patients will help determine the impact of CHIKV infection on neurocognitive outcomes during childhood. Outcomes of CHIKV infection among children from other settings have tended to range from full recovery through to neurological deficits of varying severity (especially among perinatal infections) and death [9,32].

When comparing CHIKV-positive and CHIKV-negative children, none of the clinical symptoms or laboratory tests showed differences that were substantial enough for diagnostic use in clinical practice. Diagnostic uncertainty in the absence of CHIKV RT-PCR testing (or other laboratory tests such as CHIKV IgM serology) may be clinically challenging. For instance, treating children for possible culture-negative bacterial meningitis is costly and contributes to antimicrobial resistance in a hospital setting. Neuroimaging is difficult to access in our setting and usually requires costs to be borne by parents. Capacity for definitive molecular or serological testing is therefore required for confirmatory diagnosis of a disease that appears to be a substantial cause of admission in young children in coastal Kenya.

The study has some limitations. We defined cases based on PCR detection of viral RNA, which likely underestimated the true burden of CHIKV due to the short duration of viral RNA detection in tissues during CHIKV infections [33]. Furthermore, we are likely to have underestimated more severe CHIKV infections since CSF is unlikely to be collected from the most severely unwell children. We did not screen for other viruses that have been associated with neurological disease in Africa (e.g., adenovirus, herpesvirus, and others [34]), and this warrants future study. Further studies are also needed to determine the nature and timing of mother-to-child CHIKV transmission, including maternal CHIKV screening as done by others [20]. The study was limited to a single geographical location on the Kenyan coast, and our estimate of disease incidence was based on the KHDSS population as denominator. KCH is the only secondary healthcare facility in the demographic surveillance area of Kilifi County, and KHDSS residents account for 40% to 50% of all admissions at the hospital. CHIKV infections could be detected throughout the demographic surveillance area. We excluded children from outside the demographic surveillance area from the analysis because we do not know their denominator. The denominator is well defined for those within the KHDSS. Therefore, we do not identify out-of-area admissions as a source of non-representativeness of the study. On the other hand, we recognize missed cases to lead to an underestimate of the disease incidence. We will have missed cases if they did not present to hospital (i.e., if they were self-limiting or died at home or traveled to a distant hospital outside the study area). Of these 3 possibilities, self-limiting cases would not be relevant to our case definition, and few would be likely to bypass KCH to travel elsewhere. However, deaths prior to hospital admission or healthcare are common, and, furthermore, we identify a CSF sampling bias toward less severe cases in hospital. The incidence we measure is therefore likely a minimum estimate. Future studies in other health facilities along the East African coast will help determine the generalizability of our observations.

In conclusion, we have uncovered a high burden of CHIKV-associated neurological illness in Kenyan children, with disease incidence being much higher than recent estimates for bacterial meningitis and cerebral malaria. This, together with previous studies [6,8,20], support the importance of CHIKV as a neuroinfectious arbovirus. Following the reductions in severe malaria due to falling malaria transmission [23], and reductions in bacterial meningitis after vaccination [27], CHIKV may now be one of the most common causes of hospitalization with neurological disease among children aged <5 years in coastal Kenya. CHIKV mosquito vectors are widely distributed on the East African coast and reporting of cases of CHIKV infections in Africa is widespread. Despite this, surveillance for CHIKV in CSF samples has not been undertaken systematically in Africa and should now be an urgent priority to describe this previously unidentified public health burden. Such work will provide the foundation for future research on preventive and therapeutic interventions. There are currently no licensed vaccines for CHIKV infection though several vaccine candidates are under evaluation in adults and adolescents [35]. However, none have been evaluated in young children and there has never been a clinical trial of any CHIKV vaccine in Africa. Our data support an urgent need to develop and evaluate vaccines that can safely provide protection against CHIKV infection in children in Kenya and other settings in Africa where disease is endemic. The high CHIKV disease incidence observed here and in our previous study in community health facilities [14] would allow a sufficiently powered Phase III clinical trial to evaluate vaccine efficacy and inform the unmet need for an effective control intervention against CHIKV infections. An assessment of the effectiveness of supportive treatment on neurological outcomes is also feasible given the high disease burden. Determining the pathophysiological mechanisms underlying the age-related risk (including older adults [6]) of CHIKV-associated neurological disease remains a major research priority. Such studies may identify critical host–virus interactions that could be exploited for novel antiviral treatments.

## Supporting information

S1 STROBE Checklist STROBE Statement—Checklist of items that should be included in reports of *cohort studies*. (DOCX)

S1 Table Missing data. The number and percentage of children missing data within each clinical category is shown for the respective variables included in the analysis presented in Table 1. CHIKV, chikungunya virus; CSF, cerebrospinal fluid; HIV, human immunodeficiency virus; Hb, hemoglobin; WBC, white blood cell count.
(DOCX)

S2 Table Demographic and clinical features of patients aged <3 months screened for CHIKV infection. **#**Symptoms are not mutually exclusive; some patients had overlaps in symptoms. *Refers to at least 1 seizure in the last 24 hours. **†**Sample sizes for each variable do not always add up to the total number (N) for each group due to missing data. Analysis was only performed in those with data available. Missing data are summarized in S1 Table. *P* values are from chi-squared test comparing variables, except duration of hospitalization for which a Mann–Whitney U test was used. CHIKV, chikungunya virus; CSF, cerebrospinal fluid; Hb, hemoglobin; HIV, human immunodeficiency virus; IQR, interquartile range; WBC, white blood cell count.
(DOCX)

S3 Table CHIKV incidence rates from a sensitivity analysis with 2 considerations for the at-risk population with regard to out-migration and in-migration into the study area. Denominator A excludes all the time in a migration episode. A migration episode is the duration of time between an out-migration and a subsequent in-migration. Denominator B excludes only migration episodes greater than 120 days. Therefore, all migration episodes that are less than 120 days are included in the PYO. The rationale of including episodes shorter 120 days (same as the length of one enumeration round) is that it is short enough to be considered as a migration within the study area (KHDSS)—for instance, the person moved homesteads but is still a resident in the study area, hence still at risk. The net effect of denominator B is increased PYOs resulting in slightly lower incidence estimates. CHIKV, chikungunya virus; KHDSS, Kilifi Health and Demographic Surveillance System; PYO, person-years of observation.
(DOCX)

S1 Fig Distribution of clinical indications for CSF collection. The distribution of clinical indications for lumbar puncture among all 4,332 admissions that had CSF collected during the study period are shown in panel A. In panel B, the distribution of clinical indications for the 367 children whose CSF were CHIKV positive is shown for comparison. The data are shown as percentages of the respective denominator (4,332 in A and 367 in B). The indications are organized according to a hierarchy, where we report the strongest indication for CSF collection taking the order of importance from top to bottom as: meningism; coma (BCS <3); impaired consciousness (BCS 3 or 4); seizures; prostration; fever; and other causes. Error bars represent 95% confidence intervals. BCS, Blantyre Coma Score; CHIKV, chikungunya virus; CSF, cerebrospinal fluid.
(TIFF)

S2 Fig Annual distribution of admissions at KCH stratified by clinical indication for CSF collection. The distribution of all 18,341 children aged <16 years admitted at KCH during the study period is shown, stratified by whether CSF was collected or not. The stacked bars for each clinical indication show the proportions whose CSF was collected or not collected and add up to 100% in each instance. The total number of admissions with each clinical indication over the 5-year study duration was: meningism (*n =* 136), coma (*n* = 2,780), impaired consciousness (*n* = 6,428), seizures (*n* = 1,524), prostration (*n* = 131), fever (*n* = 4,292), and others (*n* = 3,050). CSF, cerebrospinal fluid; KCH, Kilifi County Hospital.
(TIFF)

S3 Fig Distribution of deaths by clinical indication for CSF collection. There were 1,653 (9.0%) deaths among all 18,341 children aged <16 years admitted at KCH during the study period. All deaths among children within each clinical indication are stratified by whether or not CSF was collected. The total number of deaths within each clinical indication are: meningism (*n* = 24), coma (*n* = 840), impaired consciousness (*n* = 510), seizures (*n* = 20), prostration (*n* = 15), fever (*n* = 169), and others (*n* = 75). Error bars represent 95% confidence intervals. CSF, cerebrospinal fluid; KCH, Kilifi County Hospital.
(TIFF)

S4 Fig Confirmation of specificity of CHIKV RT-PCR assay used. The CHIKV RT-PCR assay used has previously been shown to be highly specific. We further confirmed its specificity for CSF samples by sequencing a random 7 RT-PCR-positive CSF samples from our study. Briefly, the sequencing protocol involved viral RNA isolation, RT-PCR amplification using a set of 41 primer pairs spanning the CHIKV genome designed using Primal Scheme (https://primalscheme.com/), amplicons visualized on gel electrophoresis, cleaned and sequenced on the Oxford Nanopore GridION using the LSK109 protocol with native barcoding (https://store.nanoporetech.com/ligation-sequencing-kit.html). The obtained sequence reads were then mapped onto a reference CHIKV genome as shown above. We included 6 RT-PCR-negative CSF samples as negative controls, and cultured CHIKV isolate as a positive control. All 7 RT-PCR samples had partial genomes generated, with sequence reads that mapped to various parts of the CHIKV genome (see figure). No CHIKV sequences were obtained from the RT-PCR-negative samples. CHIKV sequences were obtained from the positive control, as expected (data not shown). These results support the specificity and validity of our RT-PCR assay in detection of CHIKV in CSF samples. The low success in obtaining full-length genomes could be due to several reasons (as acknowledged for other arbovirus sequencing projects (e.g., [36,37]): (1) low viral load (Ct values ranged 35.2–38.0); (2) template degradation (due to RNAses or freeze–thaw cycles); or (3) sequencing primer dropouts due to competition and mutations on primer binding sites. More comprehensive CHIKV genome sequencing and phylogenetic analysis of samples from different time points, disease severity, and geographic locations is planned as a future project. CHIKV, chikungunya virus; CSF, cerebrospinal fluid; Ct, cycle threshold; RT-PCR, reverse transcriptase polymerase chain reaction.
(PDF)

## Abbreviations

BCS: Blantyre Coma Score
CHIKF: chikungunya fever
CHIKV: chikungunya virus
CSF: cerebrospinal fluid
Ct: cycle threshold
HDU: high dependency unit
IgG: immunoglobulin G
KCH: Kilifi County Hospital
KHDSS: Kilifi Health and Demographic Surveillance System
nsP1: nonstructural protein 1
PYO: person-years of observation
RT-PCR: reverse transcriptase polymerase chain reaction
