# Supplementary material for: Incidence of chikungunya virus infections among Kenyan children with neurological disease, 2014–2018: A cohort study
Source: PLoS Med. 2022 May 12;19(5):e1003994. doi: 10.1371/journal.pmed.1003994 (PMC9135332; doi:10.1371/journal.pmed.1003994)
Supplement: S1 STROBE Checklist — (DOCX) [file pmed.1003994.s001.docx]

STROBE Statement—Checklist of items that should be included in reports of ***cohort studies***

|  | Item No | Recommendation | Page No |
| --- | --- | --- | --- |
| **Title and abstract** | 1 | (*a*) Indicate the study’s design with a commonly used term in the title or the abstract | Included on title |
|  |  | (*b*) Provide in the abstract an informative and balanced summary of what was done and what was found | Information included in the Abstract |
| Introduction | | | |
| Background/rationale | 2 | Explain the scientific background and rationale for the investigation being reported | Introduction section, paragraphs 1-3 |
| Objectives | 3 | State specific objectives, including any prespecified hypotheses | Introduction section, paragraphs 3 |
| Methods | | | |
| Study design | 4 | Present key elements of study design early in the paper | Methods section provides details of clinical surveillance, study population, method used to detect CHIKV infection and statistical analysis |
| Setting | 5 | Describe the setting, locations, and relevant dates, including periods of recruitment, exposure, follow-up, and data collection | Information provided under Methods section; subsections ‘Clinical surveillance’ and ‘Statistical analysis’ |
| Participants | 6 | (*a*) Give the eligibility criteria, and the sources and methods of selection of participants. Describe methods of follow-up | Information provided under Methods section; subsections ‘Clinical surveillance’ and ‘Statistical analysis’ |
|  |  | (*b*) For matched studies, give matching criteria and number of exposed and unexposed |  |
| Variables | 7 | Clearly define all outcomes, exposures, predictors, potential confounders, and effect modifiers. Give diagnostic criteria, if applicable | Information provided under Methods section; subsections ‘Detection of CHIKV infection’ and ‘Statistical analysis’ |
| Data sources/ measurement | 8* | For each variable of interest, give sources of data and details of methods of assessment (measurement). Describe comparability of assessment methods if there is more than one group | Information provided under Methods section; subsections ‘Detection of CHIKV infection’ and ‘Statistical analysis’ |
| Bias | 9 | Describe any efforts to address potential sources of bias | Information provided under Methods section; subsection ‘Statistical analysis’ |
| Study size | 10 | Explain how the study size was arrived at | Information provided under Methods section, second paragraph of ‘Clinical surveillance’ subsection. |
| Quantitative variables | 11 | Explain how quantitative variables were handled in the analyses. If applicable, describe which groupings were chosen and why | Information provided under Methods section; subsection ‘Statistical analysis’ |
| Statistical methods | 12 | (*a*) Describe all statistical methods, including those used to control for confounding | Information provided under Methods section; subsection ‘Statistical analysis’ |
|  |  | (*b*) Describe any methods used to examine subgroups and interactions |  |
|  |  | (*c*) Explain how missing data were addressed |  |
|  |  | (*d*) If applicable, explain how loss to follow-up was addressed |  |
|  |  | (*e*) Describe any sensitivity analyses |  |
| Results | | |  |
| Participants | 13* | (a) Report numbers of individuals at each stage of study—eg numbers potentially eligible, examined for eligibility, confirmed eligible, included in the study, completing follow-up, and analysed | Information provided under Results section; paragraphs 1 and 2. Flow diagram included as Fig 1. |
|  |  | (b) Give reasons for non-participation at each stage |  |
|  |  | (c) Consider use of a flow diagram |  |
| Descriptive data | 14* | (a) Give characteristics of study participants (eg demographic, clinical, social) and information on exposures and potential confounders | Information provided under Results section; paragraphs 1 and 2, and on Tables 1 and 2, and S2 Table and S3 Table. Flow diagram included as Fig 1. Missing data presented in S1 Table. |
|  |  | (b) Indicate number of participants with missing data for each variable of interest |  |
|  |  | (c) Summarise follow-up time (eg, average and total amount) |  |
| Outcome data | 15* | Report numbers of outcome events or summary measures over time | Information presented in Tables 1, 2, S2 Table, S3 Table and Fig 1, Fig 2, S1 Fig, S2 Fig and S3 Fig |

| Main results | 16 | (*a*) Give unadjusted estimates and, if applicable, confounder-adjusted estimates and their precision (eg, 95% confidence interval). Make clear which confounders were adjusted for and why they were included | Information presented in Tables 1, 2, S2 Table, S3 Table and Fig 1, Fig 2, S1 Fig, S2 Fig and S3 Fig  Additional information also presented under ‘Results’ section, paragraphs 2-5. |
| --- | --- | --- | --- |
|  |  | (*b*) Report category boundaries when continuous variables were categorized |  |
|  |  | (*c*) If relevant, consider translating estimates of relative risk into absolute risk for a meaningful time period |  |
| Other analyses | 17 | Report other analyses done—eg analyses of subgroups and interactions, and sensitivity analyses | Sensitivity analysis of impact of migration on incidence estimates presented in S3 Table |
| Discussion | | | |
| Key results | 18 | Summarise key results with reference to study objectives | Presented under paragraph 1 of ‘Discussion’ section. |
| Limitations | 19 | Discuss limitations of the study, taking into account sources of potential bias or imprecision. Discuss both direction and magnitude of any potential bias | Presented under paragraph 2, 3 and 4 of ‘Discussion’ section. |
| Interpretation | 20 | Give a cautious overall interpretation of results considering objectives, limitations, multiplicity of analyses, results from similar studies, and other relevant evidence | Presented in the last paragraph of ‘Discussion’ section. |
| Generalisability | 21 | Discuss the generalisability (external validity) of the study results | Presented in paragraph 4 and 5 of ‘Discussion’ section. |
| Other information | | | |
| Funding | 22 | Give the source of funding and the role of the funders for the present study and, if applicable, for the original study on which the present article is based | Presented under Financial Disclosures section of the manuscript submission system |
