# Supplementary material for: Incidence of chikungunya virus infections among Kenyan children with neurological disease, 2014–2018: A cohort study
Source: PLoS Med. 2022 May 12;19(5):e1003994. doi: 10.1371/journal.pmed.1003994 (PMC9135332; doi:10.1371/journal.pmed.1003994)
Supplement: S1 Table — The number and percentage of children missing data within each clinical category is shown for the respective variables included in the analysis presented in Table 1. CHIKV, chikungunya virus; CSF, cerebrospinal fluid; HIV, human immunodeficiency virus; Hb, hemoglobin; WBC, white blood cell count. (DOCX) [file pmed.1003994.s002.docx]

**S1 Table: Missing data**

|  | **CHIKV positive**  **(N=367)** | **CHIKV negative**  **(N=3613)** |
| --- | --- | --- |
| **Laboratory investigations** | **Number missing (%)** | **Number missing (%)** |
| CSF-to-blood glucose ratio <0.67 | 154 (42.0) | 1244 (34.4) |
| CSF protein > 0.45g/L | 15 (4.1) | 161 (4.5) |
| CSF Leukocyte count >5/µL | 14 (3.8) | 119 (3.3) |
| CSF turbidity | 36 (9.8) | 312 (8.6) |
| HIV positive | 83 (22.6) | 768 (21.2) |
| Bacteraemia | 4 (1.1) | 28 (0.8) |
| Malaria slide positive | 2 (0.5) | 20 (0.6) |
| Malaria parasite density (>2500/µL) | 2 (0.5) | 20 (0.6) |
| Impaired renal function (creatinine >80µmol/L) | 31 (8.4) | 470 (13.0) |
| Severe anemia (Hb <5g/dL) | 5 (1.4) | 50 (1.4) |
| Hypoglycemia (blood glucose <2.2mmol/l) | 145 (39.5) | 1166 (32.3) |
| Thrombocytopenia (platelets <159 x10^3^/µL) | 5 (1.4) | 50 (1.4) |
| Leukopenia (WBC count<3.9 x10^3^/µL) | 5 (1.4) | 48 (1.3) |
| Lymphopenia (Lymphocyte count<1.7 x10^3^/µL) | 5 (1.4) | 51 (1.4) |
