# Supplementary material for: Incidence of chikungunya virus infections among Kenyan children with neurological disease, 2014–2018: A cohort study
Source: PLoS Med. 2022 May 12;19(5):e1003994. doi: 10.1371/journal.pmed.1003994 (PMC9135332; doi:10.1371/journal.pmed.1003994)
Supplement: S2 Table — #Symptoms are not mutually exclusive; some patients had overlaps in symptoms. *Refers to at least 1 seizure in the last 24 hours. †Sample sizes for each variable do not always add up to the total number (N) for each group due to missing data. Analysis was only performed in those with data available. Missing data are summarized in S1 Table. P values are from chi-squared test comparing variables, except duration of hospitalization for which a Mann–Whitney U test was used. CHIKV, chikungunya virus; CSF, cerebrospinal fluid; Hb, hemoglobin; HIV, human immunodeficiency virus; IQR, interquartile range; WBC, white blood cell count. (DOCX) [file pmed.1003994.s003.docx]

**S2 Table: Demographic and clinical features of patients aged <3 months screened for CHIKV infection**

|  | **CHIKV positive**  **(N=148)** | **CHIKV negative**  **(N=1641)** | **P value** |
| --- | --- | --- | --- |
| **Sex – no. (%)** |  |  | 0.84 |
| Female | 64 (43.2) | 696 (42.4) |  |
| **Age group – no. (%)** |  |  | 0.67 |
| Day of birth | 29 (19.6) | 305 (18.6) |  |
| 1 to 7 days | 88 (59.5) | 930 (56.7) |  |
| 8 to 14 days | 8 (5.4) | 134 (8.2) |  |
| 15 to 28 days | 9 (6.1) | 132 (8.0) |  |
| 29 to 90 days | 14 (9.5) | 140 (8.5) |  |
| **Year of admission – no. (%)** |  |  | <0.001 |
| 2014 | 27 (18.2) | 336 (20.5) |  |
| 2015 | 32 (21.6) | 387 (23.6) |  |
| 2016 | 64 (43.2) | 354 (21.6) |  |
| 2017 | 17 (11.5) | 248 (15.1) |  |
| 2018 | 8 (5.4) | 316 (19.3) |  |
| **Season – no. (%)** |  |  | 0.10 |
| Jan - Mar | 42 (28.4) | 489 (29.8) |  |
| Apr - Jun | 57 (38.5) | 479 (29.2) |  |
| Jul - Sep | 24 (16.2) | 318 (19.4) |  |
| Oct - Dec | 25 (16.9) | 355 (21.6) |  |
| **Perinatal history – no. (%)** |  |  |  |
| Premature birth | 15 (10.8) | 186 (12.0) | 0.68 |
| Perinatal admission | 53 (38.1) | 519 (33.4) | 0.26 |
| Unable to breastfeed | 45 (32.4) | 560 (36.1) | 0.38 |
| Cried at birth | 109 (78.4) | 1120 (72.1) | 0.11 |
| Perinatal jaundice | 11 (7.9) | 146 (9.4) | 0.56 |
| Low birth weight (<1500g) | 5 (3.6) | 66 (4.2) | 0.71 |
| **Admission characteristics** |  |  |  |
| Duration (days) of hospitalization (median, IQR) | 4 (2, 7) | 4 (3, 8) | 0.39 |
| Needed blood transfusion (no., %) | 5 (3.4) | 68 (4.2) | 0.67 |
| **General symptoms – no. (%)** |  |  |  |
| Fever | 93 (62.8) | 984 (60.0) | 0.49 |
| Vomiting | 6 (4.0) | 69 (4.2) | 0.93 |
| Diarrhea | 1 (0.7) | 17 (1.0) | 0.67 |
| Jaundice | 19 (12.8) | 274 (16.7) | 0.22 |
| Irritability | 12 (8.1) | 168 (10.2) | 0.41 |
| Rash | 1 (0.7) | 14 (0.8) | 0.82 |
| Lymphadenopathy | 0 (0) | 1 (0.1) | 0.76 |
| **Neurological symptoms – no. (%)#** |  |  |  |
| Seizures during current illness* | 22 (14.9) | 211 (12.9) | 0.19 |
| Bulging fontanelle | 7 (4.7) | 30 (1.8) | 0.02 |
| Coma | 31 (20.9) | 320 (19.5) | 0.67 |
| **Laboratory investigations – no. (%)^†^** |  |  |  |
| CSF-to-blood glucose ratio <0.67 | 28 (35.4) | 265 (26.4) | 0.08 |
| CSF protein > 0.45g/L | 119 (83.8) | 1266 (82.4) | 0.67 |
| CSF Leukocyte count >5/µL | 36 (25.3) | 349 (22.2) | 0.38 |
| CSF turbidity | 7 (5.5) | 100 (7.1) | 0.50 |
| HIV positive | 4 (3.3) | 32 (2.4) | 0.52 |
| Bacteraemia | 8 (5.4) | 102 (6.3) | 0.68 |
| Malaria slide positive | 0 (0) | 4 (0.2) | 0.55 |
| Hypoglycemia (blood glucose <2.2mmol/l) | 11 (13.2) | 210 (19.9) | 0.14 |
| Thrombocytopenia (platelets <159 x10^3^/µL) | 16 (11.0) | 195 (12.1) | 0.70 |
| Leukopenia (WBC count<3.9 x10^3^/µL) | 1 (0.7) | 20 (1.2) | 0.56 |
| Lymphopenia (Lymphocyte count<1.7 x10^3^/µL) | 2 (1.4) | 32 (2.0) | 0.61 |
