# Supplementary material for: Incidence of chikungunya virus infections among Kenyan children with neurological disease, 2014–2018: A cohort study
Source: PLoS Med. 2022 May 12;19(5):e1003994. doi: 10.1371/journal.pmed.1003994 (PMC9135332; doi:10.1371/journal.pmed.1003994)
Supplement: S3 Table — Denominator A excludes all the time in a migration episode. A migration episode is the duration of time between an out-migration and a subsequent in-migration. Denominator B excludes only migration episodes greater than 120 days. Therefore, all migration episodes that are less than 120 days are included in the PYO. The rationale of including episodes shorter 120 days (same as the length of one enumeration round) is that it is short enough to be considered as a migration within the study area (KHDSS)—for instance, the person moved homesteads but is still a resident in the study area, hence still at risk. The net effect of denominator B is increased PYOs resulting in slightly lower incidence estimates. CHIKV, chikungunya virus; KHDSS, Kilifi Health and Demographic Surveillance System; PYO, person-years of observation. (DOCX) [file pmed.1003994.s004.docx]

**S3 Table: CHIKV incidence rates from a sensitivity analysis with two considerations for the at-risk population with regard to out-migration and in-migration into the study area**

|  | **Denominator option A** | | **Denominator option B** | |
| --- | --- | --- | --- | --- |
| **Year** | **n/PYO** | **Incidence/100,000 (95% CI)** | **n/PYO** | **Incidence /100,000 (95% CI)** |
| 2014 | 41/138881 | 29.52 (21.7-40.1) | 41/140128 | 29.3 (21.5-39.7) |
| 2015 | 50/138008 | 36.2 (27.5-47.8) | 50/139159 | 35.9 (27.2-47.4) |
| 2016 | 79/136770 | 57.8 (46.3-72) | 79/139307 | 56.7 (45.5-70.7) |
| 2017 | 18/138861 | 13 (8.2-20.6) | 18/140233 | 12.8 (8.1-20.4) |
| 2018 | 19/139067 | 13.7 (8.7-21.4) | 19/140022 | 13.6 (8.7-21.3) |
| 2014-2018 | 207/691587 | 29.9 (26.1-34.3) | 207/698848 | 29.6 (25.8-33.9) |
