# Supplementary figures and images for: Incidence of chikungunya virus infections among Kenyan children with neurological disease, 2014–2018: A cohort study
Source: PLoS Med. 2022 May 12;19(5):e1003994. doi: 10.1371/journal.pmed.1003994 (PMC9135332; doi:10.1371/journal.pmed.1003994)

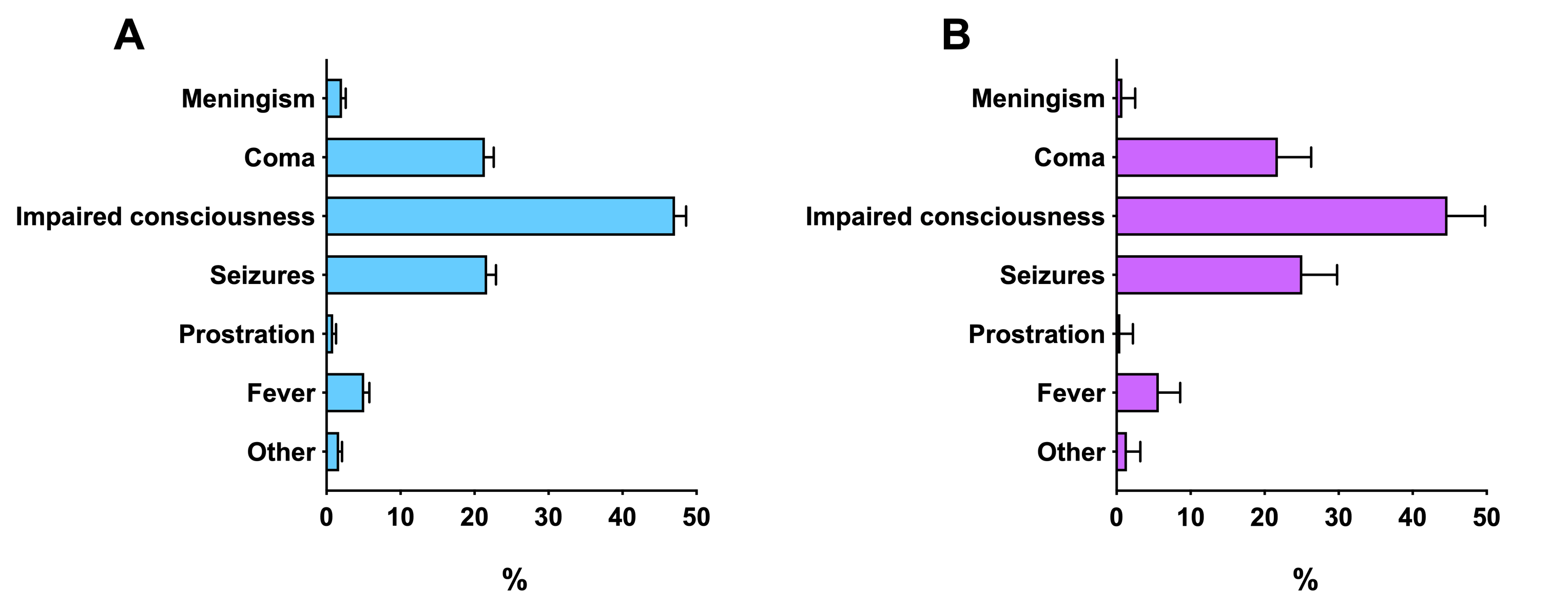

Supplement: S1 Fig — The distribution of clinical indications for lumbar puncture among all 4,332 admissions that had CSF collected during the study period are shown in panel A. In panel B, the distribution of clinical indications for the 367 children whose CSF were CHIKV positive is shown for comparison. The data are shown as percentages of the respective denominator (4,332 in A and 367 in B). The indications are organized according to a hierarchy, where we report the strongest indication for CSF collection taking the order of importance from top to bottom as: meningism; coma (BCS <3); impaired consciousness (BCS 3 or 4); seizures; prostration; fever; and other causes. Error bars represent 95% confidence intervals. BCS, Blantyre Coma Score; CHIKV, chikungunya virus; CSF, cerebrospinal fluid. (TIFF) [file pmed.1003994.s005.tiff]

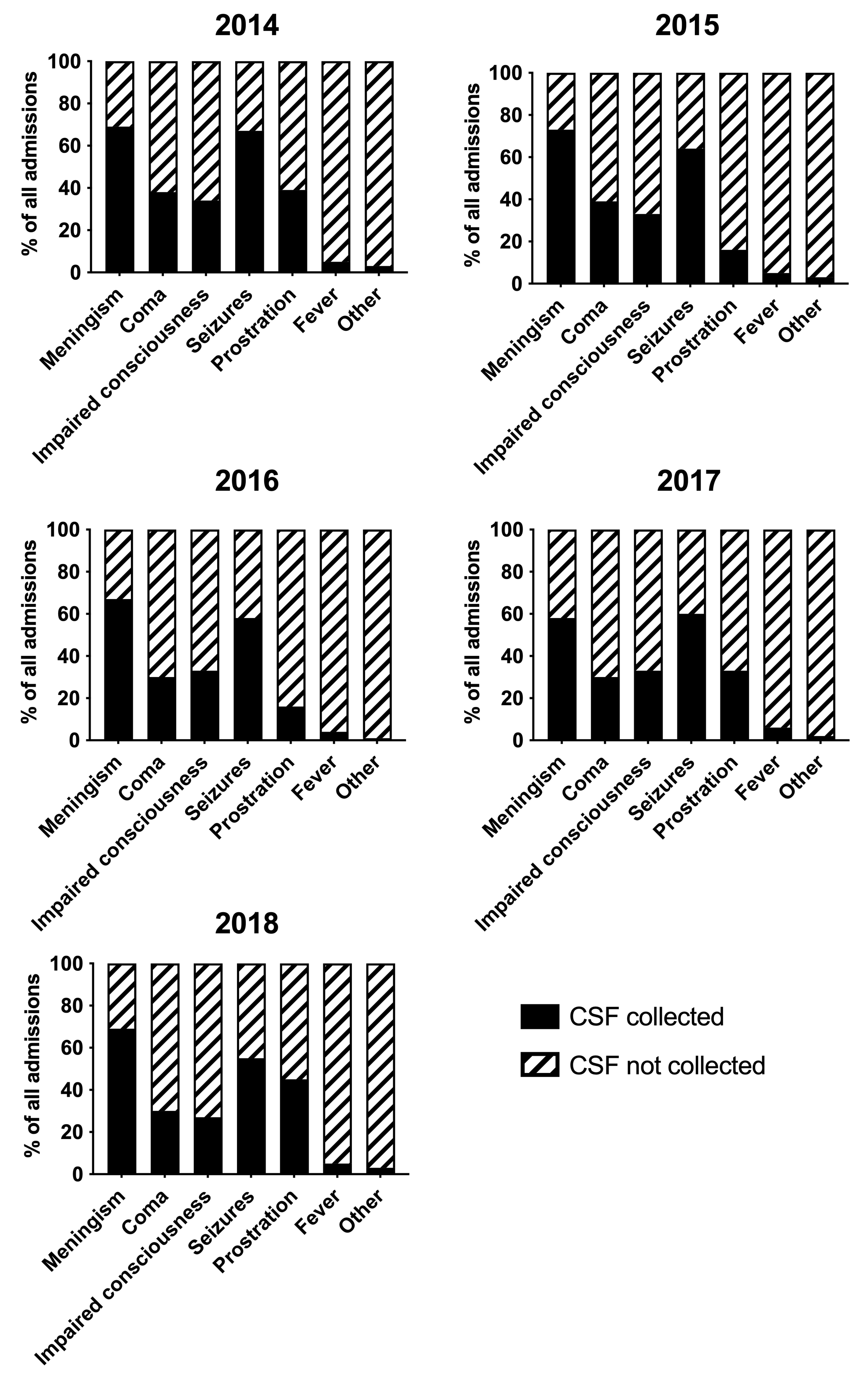

Supplement: S2 Fig — The distribution of all 18,341 children aged <16 years admitted at KCH during the study period is shown, stratified by whether CSF was collected or not. The stacked bars for each clinical indication show the proportions whose CSF was collected or not collected and add up to 100% in each instance. The total number of admissions with each clinical indication over the 5-year study duration was: meningism (n = 136), coma (n = 2,780), impaired consciousness (n = 6,428), seizures (n = 1,524), prostration (n = 131), fever (n = 4,292), and others (n = 3,050). CSF, cerebrospinal fluid; KCH, Kilifi County Hospital. (TIFF) [file pmed.1003994.s006.tiff]

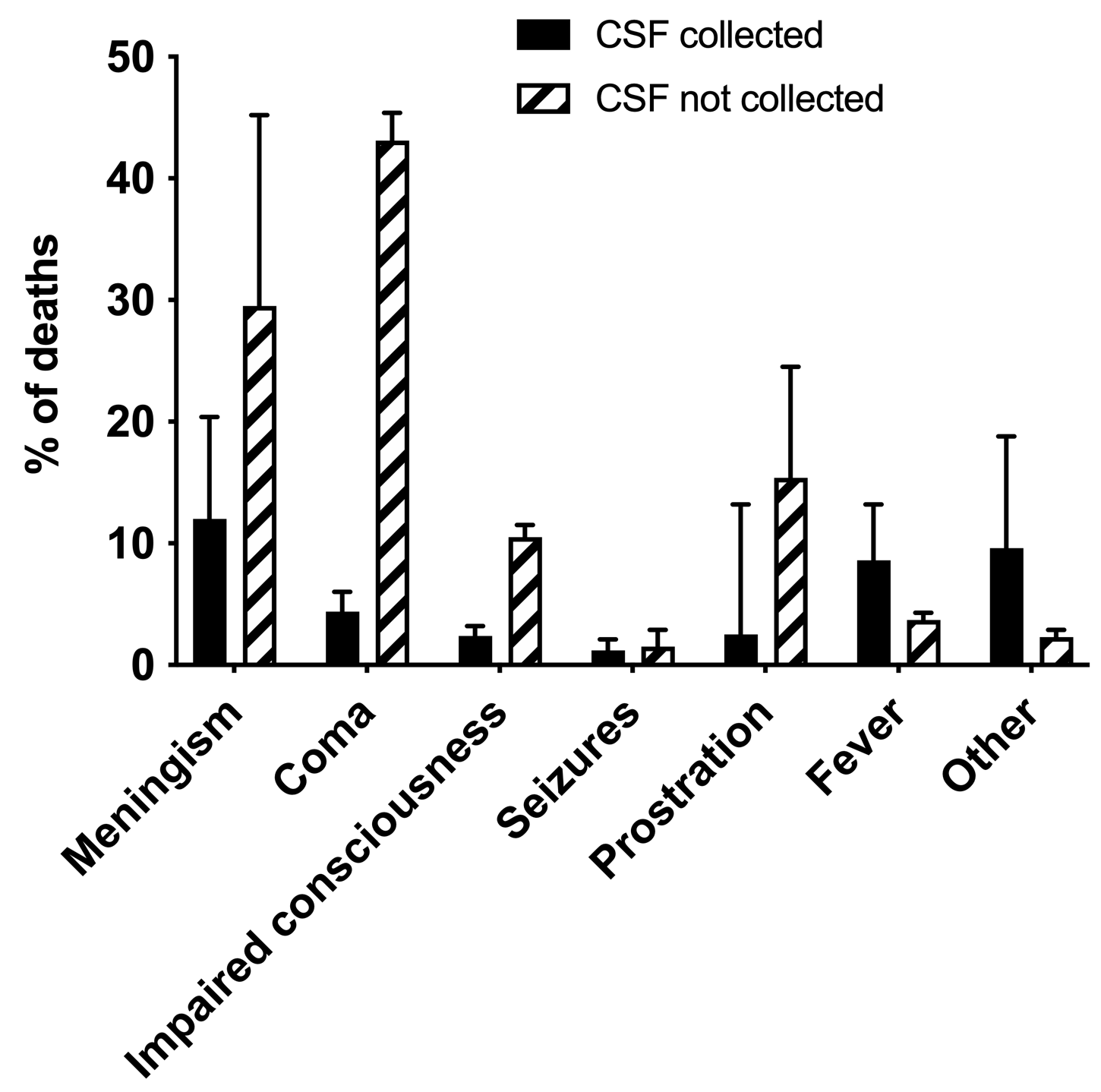

Supplement: S3 Fig — There were 1,653 (9.0%) deaths among all 18,341 children aged <16 years admitted at KCH during the study period. All deaths among children within each clinical indication are stratified by whether or not CSF was collected. The total number of deaths within each clinical indication are: meningism (n = 24), coma (n = 840), impaired consciousness (n = 510), seizures (n = 20), prostration (n = 15), fever (n = 169), and others (n = 75). Error bars represent 95% confidence intervals. CSF, cerebrospinal fluid; KCH, Kilifi County Hospital. (TIFF) [file pmed.1003994.s007.tiff]
