# Supplementary material for: Incidence of chikungunya virus infections among Kenyan children with neurological disease, 2014–2018: A cohort study
Source: PLoS Med. 2022 May 12;19(5):e1003994. doi: 10.1371/journal.pmed.1003994 (PMC9135332; doi:10.1371/journal.pmed.1003994)

CSF samples reads mapped on to CHIKV reference (HQ456255.1)

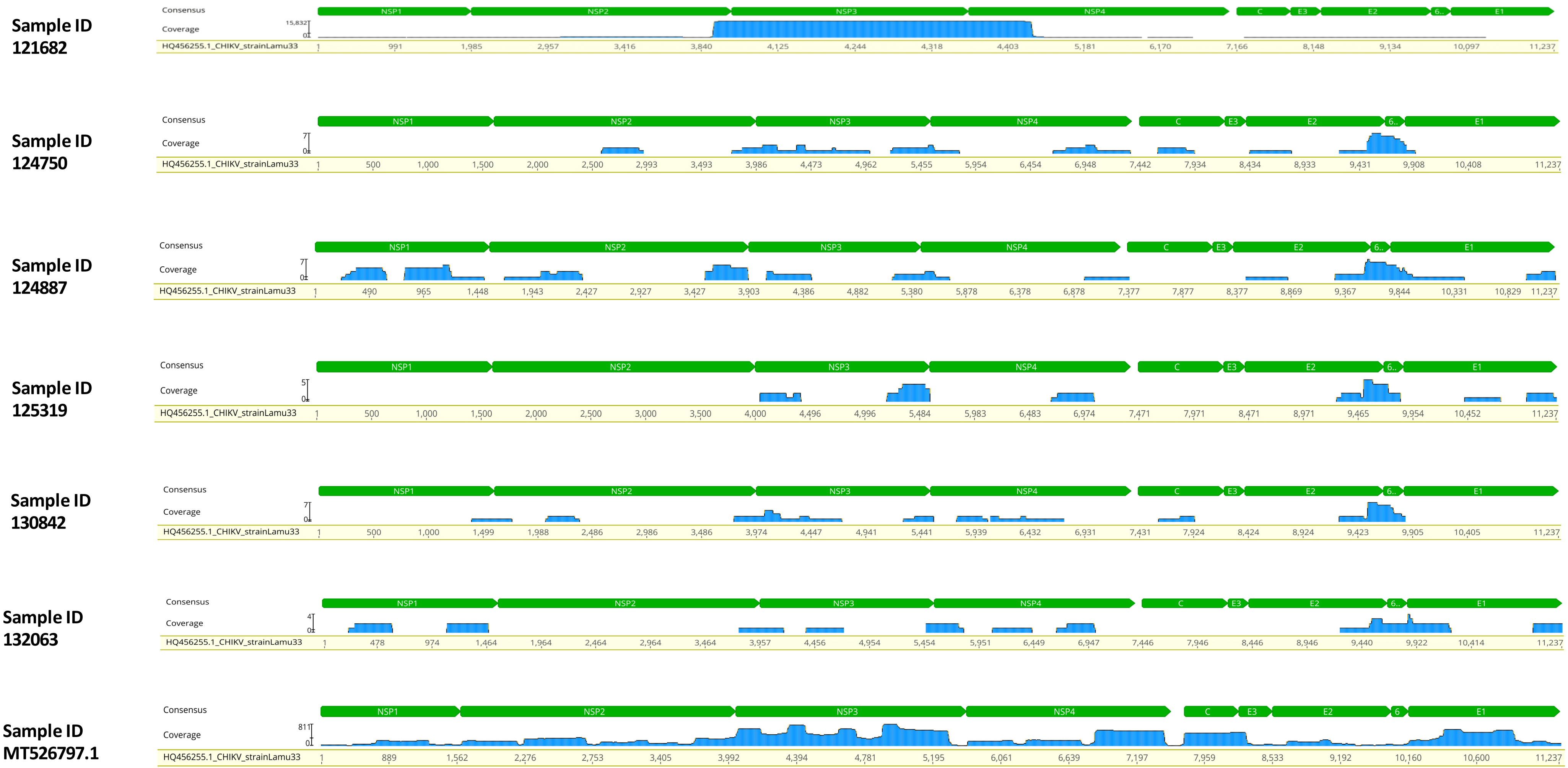

Supplement: S4 Fig — The CHIKV RT-PCR assay used has previously been shown to be highly specific. We further confirmed its specificity for CSF samples by sequencing a random 7 RT-PCR-positive CSF samples from our study. Briefly, the sequencing protocol involved viral RNA isolation, RT-PCR amplification using a set of 41 primer pairs spanning the CHIKV genome designed using Primal Scheme (https://primalscheme.com/), amplicons visualized on gel electrophoresis, cleaned and sequenced on the Oxford Nanopore GridION using the LSK109 protocol with native barcoding (https://store.nanoporetech.com/ligation-sequencing-kit.html). The obtained sequence reads were then mapped onto a reference CHIKV genome as shown above. We included 6 RT-PCR-negative CSF samples as negative controls, and cultured CHIKV isolate as a positive control. All 7 RT-PCR samples had partial genomes generated, with sequence reads that mapped to various parts of the CHIKV genome (see figure). No CHIKV sequences were obtained from the RT-PCR-negative samples. CHIKV sequences were obtained from the positive control, as expected (data not shown). These results support the specificity and validity of our RT-PCR assay in detection of CHIKV in CSF samples. The low success in obtaining full-length genomes could be due to several reasons (as acknowledged for other arbovirus sequencing projects (e.g., [36,37]): (1) low viral load (Ct values ranged 35.2–38.0); (2) template degradation (due to RNAses or freeze–thaw cycles); or (3) sequencing primer dropouts due to competition and mutations on primer binding sites. More comprehensive CHIKV genome sequencing and phylogenetic analysis of samples from different time points, disease severity, and geographic locations is planned as a future project. CHIKV, chikungunya virus; CSF, cerebrospinal fluid; Ct, cycle threshold; RT-PCR, reverse transcriptase polymerase chain reaction. (PDF) [file pmed.1003994.s008.pdf]
